# Coffee Waste Macro-Particle Enhancement in Biopolymer Materials for Edible Packaging

**DOI:** 10.3390/polym15020365

**Published:** 2023-01-10

**Authors:** Samsul Rizal, H.P.S. Abdul Khalil, Shazlina Abd Hamid, Ikramullah Ikramullah, Rudi Kurniawan, Che Mohamad Hazwan, Umar Muksin, Sri Aprilia, Tata Alfatah

**Affiliations:** 1 Department of Mechanical Engineering, Universitas Syiah Kuala, Banda Aceh 23111, Indonesia; 2 Bioresource Technology Division, School of Industrial Technology, Universiti Sains Malaysia, Penang 11800, Malaysia; 3 Green Biopolymer, Coatings and Packaging Cluster, School of Industrial Technology, Universiti Sains Malaysia, Penang 11800, Malaysia; 4 Department of Physics, Universitas Syiah Kuala, Banda Aceh 23111, Indonesia; 5 Department of Chemical Engineering, Universitas Syiah Kuala, Darussalam, Banda Aceh 23111, Indonesia

**Keywords:** coffee waste, seaweed, biopolymer film, edible film, food packaging

## Abstract

Plastic pollution has raised interest in biodegradable and sustainable plastic alternatives. For edible food packaging, seaweed biopolymers have been studied for their film-forming properties. In this study, packaging films were developed using the solvent casting technique from natural red seaweed (*Kappaphycus alvarezii*) and coffee waste product. The physico-chemical and thermal properties of seaweed/coffee biopolymer films was obtained using dynamic light scattering (DLS), scanning electron microscopy (SEM), Fourier transmission irradiation (FT-IR), water contact angle measurement (WCA) and thermogravimetric analysis (TGA). The characterization study was carried out to improve the film’s morphological, thermal, and mechanical properties. The average particle size of coffee waste was found to be between 1.106 and 1.281 µm, with a zeta potential value of −27.0 mV indicating the compound’s strong negative charge. The SEM analysis revealed that the coffee filler was evenly dispersed in the polymer matrix, improving the film’s structural properties. The FT-IR result shows that coffee waste was successfully incorporated over the film matrix with the presence of a N-H bond. The hydrophobic property of the film was enhanced with the incorporation of coffee filler, indicating increased water contact angle compared to the neat film. The tensile properties of the biopolymer film were significantly improved at 4 wt% coffee powder with optimum tensile strength (35.47 MPa) with the addition of coffee waste powder. The incorporation of coffee waste into the seaweed matrix increased the functional properties of the fabricated biopolymer film. Thus, seaweed/coffee biopolymer film has the potential to be used in food packaging and other applications.

## 1. Introduction

Edible films are edible packaging materials made from edible ingredients. Because of their environmental friendliness, safety of consumption, and ease of use, edible films are becoming increasingly popular [1]. This is because edible films can improve food quality, freshness, and shelf-life. The edible films form a semipermeable barrier over the packaged food product, which improves its barrier properties by reducing moisture, lipid, gas, and volatile exchange [2,3]. Biopolymers have been studied for their film-forming capabilities in order to create edible films for food packaging [4]. Seaweed is a particularly promising renewable biopolymer source [5]. Seaweed polysaccharide-derived biopolymers have promising properties in that they are renewable, biodegradable, biocompatible, and environmentally friendly. These biopolymers have demonstrated excellent attributes for a variety of applications due to their unique film-forming ability and excellent mechanical properties. Furthermore, seaweed has flexibility and transparency properties that will aid in the development of edible films [6].

Various modification techniques, such as reinforcement and blending, can be used to improve the properties of seaweed films [7]. Seaweeds are mixed with other polysaccharides, nanoparticles, essential oils, or plant extracts to improve their barrier, mechanical, thermal, antioxidant, and antibacterial properties [8]. In previous research, biopolymers such as chitosan were combined with seaweeds to form an edible film. Albertos et al. [8] created an edible film made of seaweed (*Himanthalia elongate* and *Palmaria palmata*) and chitosan that increases the shelf-life of fish burgers by reducing microbial growth and lipid oxidation. Furthermore, chitosan, alginate, and fucoidan were used to create an edible seaweed film [9]. Gomaa et al. [9] discovered that combining these materials improves the barrier properties and antioxidant properties of the film. Oyenkami et al. [10] created a seaweed *(Euchema cotonii)* film by combining cinnamon essential oil and cellulose nanofiber. The biopolymer film derived from seaweed demonstrated satisfactory mechanical and thermal stability, as well as improved surface hydrophobicity [10].

Coffee is one of the world’s most popular beverages and has been consumed for over a thousand years [9]. After processing, approximately half of the coffee beans are wasted during the preparation of a coffee beverage. The global consumption of coffee beverages has resulted in massive amounts of coffee waste. According to reports, 6 million tons of waste are generated globally each year [11]. Malaysia has a population of about 30 million people, and per capita coffee consumption is around 800 g [12]. Byproducts of green coffee processing (pulp, mucilage, parchment, and husk) and roasting (silver skin and spent coffee grounds) are produced by the coffee industry [11]. Despite their high potential value, all of these fractions are simply discarded. Coffee waste is an underutilized source of nutrients and energy due to its high concentration of organic compounds such as fatty acids, lignin, cellulose, hemicelluloses, and other polysaccharides [4]. Coffee byproduct molecules have antioxidant and antimicrobial properties, as well as surface hydrophobicity, gas permeability, and increased mechanical resistance [11]. These properties can be used to create active food packaging. Coffee waste, on the other hand, is regarded as a serious environmental and social issue when not properly managed. The presence of high-content organic material and compounds in coffee waste is harmful to the environment. As a result, coffee waste should be properly managed in order to be converted into value-added products.

The production of biopolymeric films from seaweed and coffee waste promotes the use of low-cost sources of polysaccharides while adding value to waste for food packaging materials. The potential of coffee waste as a filler in seaweed polymer film for improving the chemical, thermal, physical, and mechanical properties of the polymer matrix was investigated further in this study. The rheological analysis of a seaweed film-forming solution mixed with coffee powder was investigated using a rheometer. The surface properties of the fabricated seaweed–coffee biopolymer film, including morphology, chemical and mechanical stability, were investigated using scanning electron microscopy (SEM), Fourier-transform infrared spectroscopy (FT-IR), particle size analyzer, zeta potential, and mechanical measurements according to the standard methods. The hydrophobicity and thermal properties of the biopolymer films were also assessed using contact angle measurement and thermogravimetric analysis (TGA).

## 2. Research Methodology

### 2.1. Materials

In this study, the coffee waste product was provided by the local industry in Penang, Malaysia, while the red seaweed (*Kappaphycus alvarezii* sp.) was purchased from Green Leaf Synergy Sdn. Bhd. The coffee waste was sieved (mesh size: 125 microns) and then ground with a ball mill (horizontal ball mill, fabricated, 160 rpm) to create fine powders for 72 h before use. Throughout the making of the films, distilled water and glycerol (Ajax Chemicals, Univar) were used as a solvent.

### 2.2. Preparation of Seaweed-Coffee Biopolymer Film

The solvent casting process was used in this study to create the seaweed/coffee biopolymer films. On a beaker, 10 g of finely cut red seaweed (*Kappaphycus alvarezii*) (1 cm lengths) was dissolved in 500 mL of distilled water with 5 g of glycerol (plasticizers). The coffee waste powder was added to the solution at various loadings (0%, 1%, 2%, 3%, 4%, and 5%) based on the dried weight of the seaweed (wt%). The mixture was then blended for 3 min to create a homogeneous seaweed slurry. The mixture solution was then heated for one and a half hours at 90 °C with constant stirring (500 rpm). After the heating process was completed, the hot solution was allowed to cool at room temperature for 15 min before being cast onto a casting tray and dried in a 45 °C oven for 12 h. The dried seaweed/coffee biopolymer film was peeled from the tray and immediately dried in a desiccator at 50% relative humidity for at least 48 h before further analysis and testing.

### 2.3. Characterization of Seaweed–Coffee Biopolymer Film

The functional properties of the fabricated films, which included morphological, rheological, chemical, water contact angle measurements and thermal properties, were conducted according to standard methods. The nano-particle size distribution and zeta potential for coffee waste powder were analyzed using a Malvern Zetasizer Nano ZS 7.11 (Malvern Instruments Ltd., Malvern, UK). Distilled water with a refractive index (1.330) was added as a dispersant to the sample cell, and then, the sample was sonicated for 10 min. The measurement used set parameters such as viscosity (0.8872) and material refractive indexes (1.47) and adjusted the pH value of each suspension by adding NaOH or HCl. The zeta potential of the original aqueous coffee waste suspension in 0.1 mM KCl electrolyte was calculated as a function of the concentration of the suspension.

The rheology of seaweed/coffee film solution is important in determining the final characteristics of the biopolymer film as packaging material. The rheological properties of seaweed/coffee film solutions were determined by using a rheometer (AAR1000-N, TA Instruments, New Castle, DE, USA). The rheometer was equipped with different geometries, which were flat or cone, rotating upper plates. The 2° cone upper plate with a diameter of 4 cm was used for all the tests. The steady state flow test of the film solution was conducted with the rheometer. The temperature-controlled rheometer was set at 25 °C while the gap was maintained constant (54 µm). Samples were sheared continuously for 30 min.

The surface morphology of coffee waste powder was examined using scanning electron microscopy (SEM) (FEI Quanta 450 FEG, FEI Technologies Inc., Hillsboro, OR, USA) at magnifications of 1000–3000×, whereas the surfaces of seaweed/coffee films were analyzed using scanning electron microscope (HITACHI S-3000 N, Hitachi Ltd., Tokyo, Japan) at 1000–2500× magnifications. The film samples were first prepared by drying overnight at 60 °C in an oven before testing. Before the SEM examination, film samples were tapped to the SEM pin holder with double-sided carbon adhesive tape. Then, the film samples were coated with a thin layer of gold before imaging in order to intensify their electrical conductivity.

The functional groups of waste coffee powder and seaweed/coffee biopolymer film were determined using Fourier transforms infrared spectroscopy–attenuated total reflectance (FTIR-ATR) (Perkin-Elmer, PC1600, PerkinElmer, Inc., Waltham, MA, USA) (Zinc Selenide (SeZn) Optical ATR plate) under wavenumber range of 4000 to 500 cm^−1^. Film samples were prepared by cutting into dimension (3 × 3 cm) and oven-dried in the oven at 60 °C for 24 h before FT-IR analysis.

The water contact angle (WCA) of the film was measured by a sessile drop technique using a contact angle analyzer (CAM 101, KSV Instruments Ltd., Helsinki, Finland) at room temperature after a water drop of 6 µL was placed on the surfaces of the film using a micro-syringe that operated automatically. Initially, all film samples were placed on the movable sample stage and levelled horizontally before measurement. The WCA was measured on both sides (left and right) of the drop, and their values were added up. For each film sample, five measurements were taken, and their results were averaged.

The thermal stability of the seaweed/coffee film was investigated using a Mettler-Toledo thermogravimetric analyzer model (Mettler Toledo, Schwarzenbach, Switzerland) between 30 and 800 °C at a heating rate of 10 °C/min under nitrogen atmosphere. For each analysis, about 6 mg of sample was taken in an aluminum sample cup, and the empty cup was used as a reference. The sample weight loss (%) and char residue were obtained from the TGA curve and the maximum decomposition temperature (T_max_) of samples obtained from the DTG curve.

The tensile test of seaweed/coffee biopolymer films was determined at room temperature based on ASTM D882-02 standard method using a miniature tensile tester (MTT175, Dia-Stron Ltd., Andover, UK) equipped with 5 kg load cell. Five samples (10 × 150 mm) were cut from each seaweed/coffee film using a utility knife. The samples were conditioned initially in a desiccator at 23 °C and 50% relative humidity (RH) before testing based on the standard. The initial grip separation and test speed were set at 100 mm and 100 mm/min, respectively. The tensile strength, Young’s modulus and elongation at the break of the films were evaluated.

## 3. Results and Discussion

**Figure 1:**
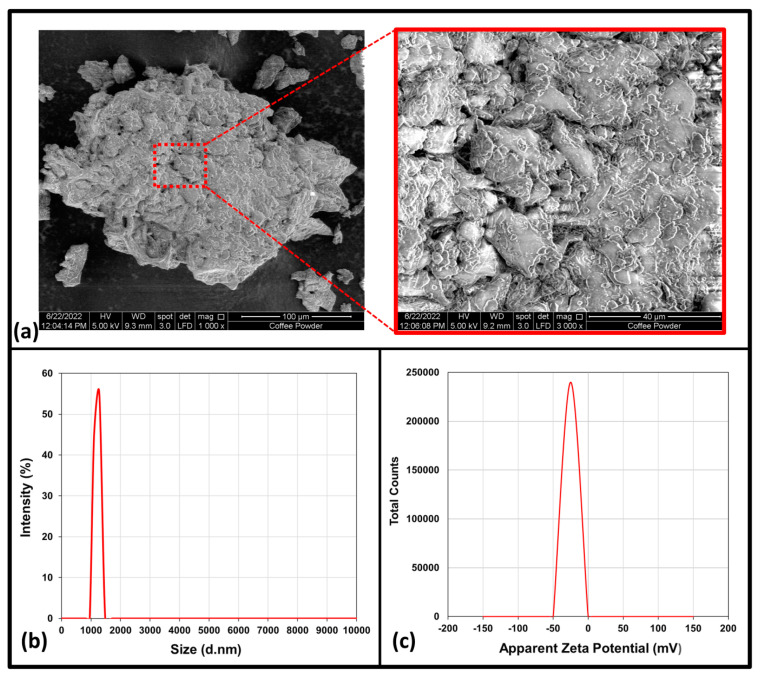
(**a**) SEM of coffee powder, (**b**) particle size distribution by intensity of coffee waste powder dispersion and (**c**) Zeta potential value of coffee waste suspension.

The surface morphology of the coffee waste powder was obtained from the SEM images. Figure 1a represents the surface appearance of coffee waste powder at 1000× and 5000× magnifications. Based on SEM examination, it was observed that the morphology of the coffee waste powder is denser, irregular, and rough. Kundasami et al. [13] discovered a similar morphology. The increased surface roughness may have a detrimental influence on the polymer’s wetting of fillers. The particles’ well-developed surface improves the contact surface between the matrix and the filler, contributing to stronger mechanical adhesion [14]. The particle size distribution of a coffee waste suspension was determined using dynamic light scattering. Figure 1b depicts a narrow particle size distribution with average diameters ranging from 1.106 to 1.281 µm and intensities ranging from 44.9% to 55.1%. These findings indicate that the majority of the coffee particle sizes are in the micro range. Thus, incorporating filler with a higher loading amount during film fabrication necessitates a greater possibility of filler agglomeration.

The electrophoretic mobility of coffee waste particles in a diluted suspension was determined using zeta potential measurements [15]. Figure 1c displays the zeta potential value of the coffee waste suspension. It shows that the coffee waste suspension has a negative value of −27.0 mV. Higher zeta potential values indicate greater dispersion capacity, while lower values indicate decreased dispersion stability [16]. This value represents a negative charge value that is close to zero. Coffee waste’s zeta potential was found to be stable because the absolute value should be greater than −25.0 mV with no tendency to flocculate [17]. This value indicates that sufficient mutual repulsion occurred, resulting in colloidal stability, which may be a prerequisite for improving the mechanical properties of a seaweed-based biopolymer film [18]. Campos et al. obtained a similar result, measuring the zeta potential of coffee waste to be −16.9 mV, which is also a negative value [15]. The coffee suspension has a high negative zeta potential due to the presence of protonated amine groups on the surface [19].

**Figure 2:**
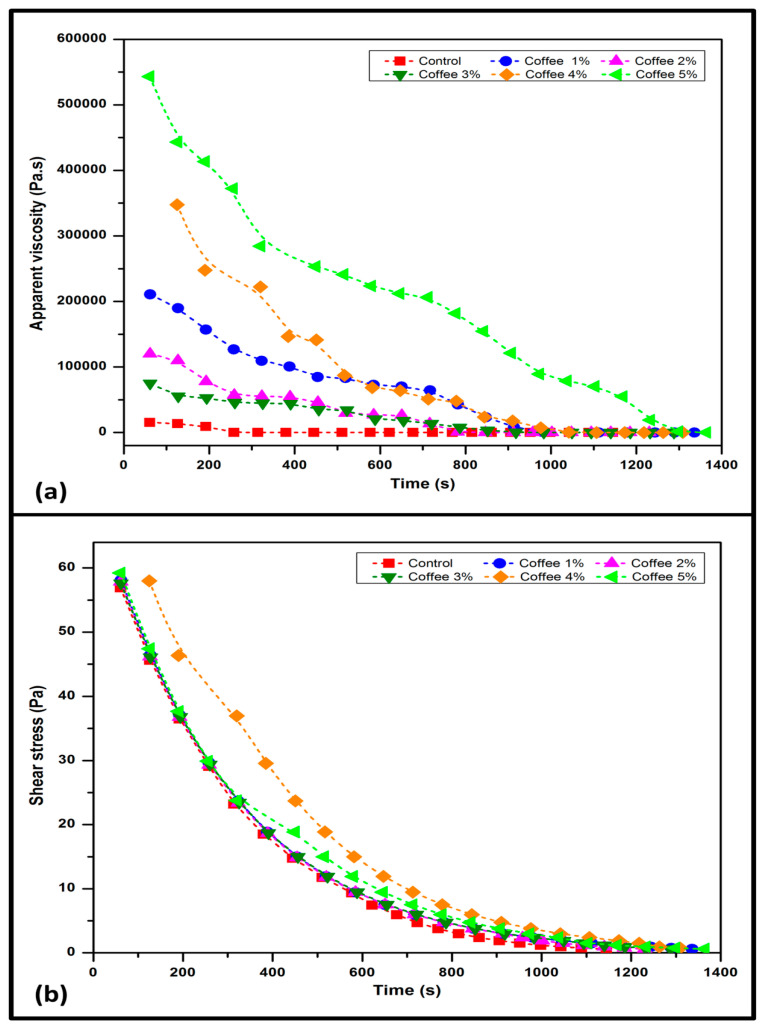
(**a**) The relation between time of shearing and apparent viscosity for seaweed/coffee film solution. (**b**) Effect of shear stress on time of seaweed/coffee film solution at different coffee loadings.

The flow and deformation of the substances were obtained from the rheological test. Figure 2a exhibits the relationship between shearing time and viscosity in seaweed/coffee solution films. Because the material is shear thinning and time dependent, the viscosity decreases continuously with time. This means that shearing the material disrupts the aggregated particles, causing them to offer less resistance to flow, and the viscosity decreases with time until it reaches a constant value. Because viscosity increased with seaweed concentration, all samples were found to be thixotropic. Thixotropy is a time-dependent property of shear thinning. When shaken, agitated, shear-stressed, or otherwise stressed, certain gels or fluids that are thick or viscous under static conditions will flow (become thinner, less viscous) over time (time-dependent viscosity) [20]. When a thixotropic material is sheared at a constant shear rate, its viscosity decreases over time, implying a progressive breakdown of structure. The viscosity does not increase consistently due to the non-uniformity of particle dispersion inside the film solution. The dispersion of particles influences the viscosity of a solution. When the volume fraction remains constant, a sample consisting of relatively large particles and a small proportion of small particles will have a lower viscosity than either large or small particles alone. Due to the competing impacts of small and large particles inside the solution, the viscosity becomes inconsistent [21].

From Figure 2b, the shear stress decreases with time during the shearing process at a constant temperature (25 °C) as the seaweed/coffee mechanically degrades and loses its consistency. The mechanical degradation is responsible for the reduction in shear stress. The structure of the seaweed/coffee in the film solution is damaged during the degradation process. This leads to a decrease in shear stress and consistency. This is an irreversible process that contributes to entropy production. The decreasing trend continues until the maximum level of damage for the applied shear rate is reached. The shear stress then becomes stable and constant. Because rheological parameters can affect the spread ability, thickness, and uniformity of the liquid coating layer during film fabrication, the rheological properties of the film-forming solution are an important study. The flow behavior of film solutions influenced the mechanical properties and optimized the processing design during application. Casting of film solution in thin layers and elimination of air bubbles will be difficult during high-shear processing operations if the viscosity is high or the structure is gel-like. Rheological parameters are also useful for evaluating the structure–function relationships of polysaccharide solution systems [22].

**Figure 3:**
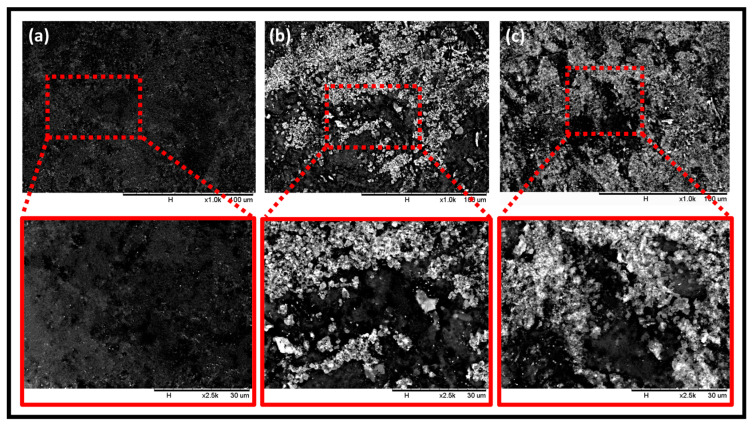
SEM images of seaweed/coffee biopolymer film at various filler loadings (**a**) control, (**b**) 1 wt% coffee and (**c**) 5 wt% coffee at 1000× and 2500× magnifications.

The surface morphology of the neat film and the seaweed/coffee-reinforced film at different concentrations of coffee filler are illustrated in Figure 3a–c. According to the figure, there was a slight difference in the morphology of the control (0 wt% coffee) and modified (1 and 5 wt% coffee) films in terms of the surface appearance of the films. The successful immobilization of the coffee particles on the seaweed biopolymer film was confirmed by SEM images. At 0 wt% coffee (control), it showed a relatively smooth surface of seaweed film. However, after the addition of coffee particles in the seaweed solution, it was found that the coffee particles appeared on the film’s surface. At high coffee loadings, more coffee particles are distributed around the surface of the film. However, the non-uniformity of dispersion of coffee waste particles was observed as the coffee concentration in the seaweed film increased from 1 to 5 wt%. This could be because the coffee particles were not well dispersed in the solution during the film’s preparation. The addition of coffee filler to the polymer matrix would significantly improve the mechanical properties of the films by reducing the porosity of the biopolymer film [23]. Aside from that, the interaction of strong intermolecular hydrogen bonding with numerous hydroxyl groups from seaweed and coffee, which have good miscibility with each other [6,24], was responsible for this enhancement. Filler agglomeration caused poor dispersion and distribution of coffee filler across the film, preventing the formation of a homogeneous mixture and lowering film performance [25]. Coffee agglomeration is important in determining the properties of biopolymer films. Because coffee has a strong tendency to agglomerate, incorporating only a small percentage of coffee is an alternative solution. Furthermore, combining low percentages of coffee loading can result in excellent dispersion and distribution. Coffee has a large interfacial area per unit volume due to its high specific surface area, which increases coffee–matrix interaction and aids in stress transfer between the coffee and the matrix. The mechanical properties of film will improve as a result of this achievement. A small amount of coffee will help to reduce the possibility of agglomeration in the polymer matrix [26]. As a result, incorporating a small percentage of coffee is an alternative solution to the problem.

**Figure 4:**
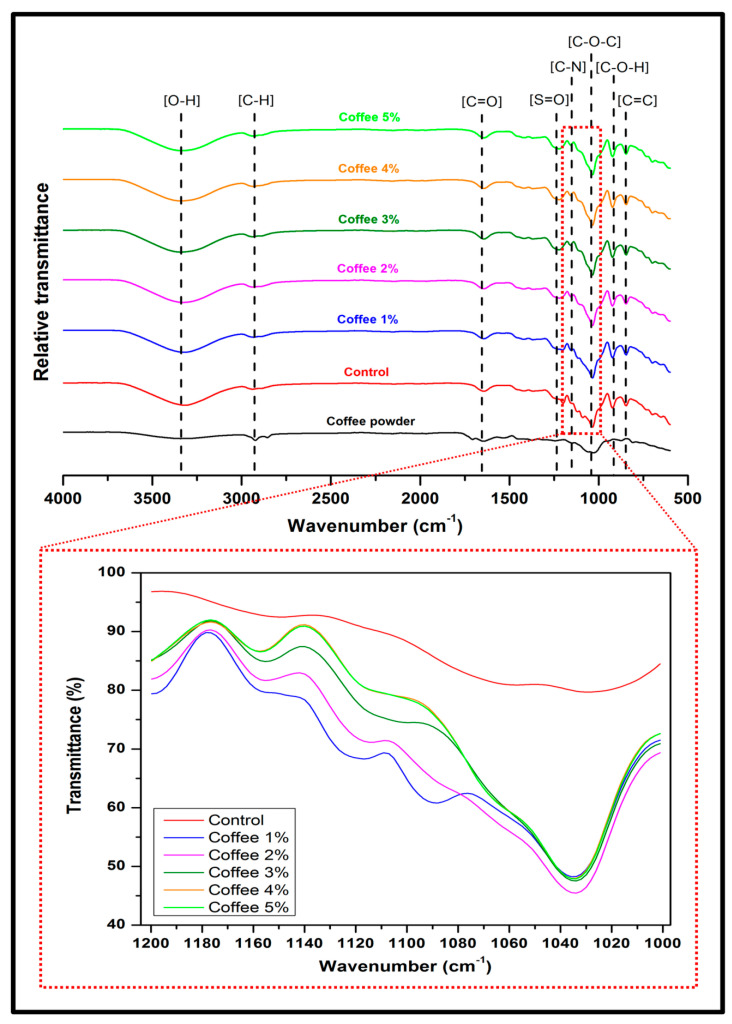
FT-IR spectra of coffee waste powder and seaweed/coffee biopolymer film at various coffee loadings.

The structural changes and the intermolecular interactions of the neat film, the seaweed/coffee film and coffee powder were obtained from the FT-IR spectra as demonstrated in Figure 4. The characteristic peaks of coffee waste powder and seaweed biopolymer film at various coffee loadings were determined by FT-IR analysis at wavenumbers 4000 to 500 cm^−1^. Coffee waste exhibited bands typical of lignocellulosic materials, as its major constituents are hemicellulose, cellulose, lignin, and other small molecules [27]. The broad band maximizing at 3332 cm^−1^ in the spectrum was related to the O-H stretching and bending vibration, which was attributed to the presence of O-H moieties in the lignocellulose of the coffee material and seaweed polysaccharides [20]. Broad and wide peaks were also observed at 3329 cm^−1^ for all films containing seaweed, which were attributed to the stretching vibration of hydroxyl (−OH) groups caused by hydrogen bond formation. The intensity and width of the overall O-H band increased significantly as the coffee was incorporated into the seaweed film. This could be due to an increase in hydrogen bonding between the seaweed and the coffee [28].

Major peaks at 2924 and 2854 cm^−1^ were observed for both coffee waste and seaweed film, indicating the presence of C-H asymmetric and symmetric stretching vibrations of CH_3_ in the cellulose. The stretching of C=O carbonyl groups is represented by the peak at 1641 cm^−1^, while the C=C vibration of lipids and aromatic rings from lignin moieties is represented by the peaks at 1527 cm^−1^. Peaks observed at 1372, 1244, and 1057 cm^−1^ represent the stretching of ester linkages found in caffeine molecules’ chlorogenic acids. Furthermore, the peak 1030 cm^−1^ indicates C-N stretching. Peaks at 1210–1220 cm^−1^ that appeared in all biopolymer films and corresponded to the stretching vibration of the S=O bond represent the characteristics of sulphate, which are commonly found in carrageenan types [29]. The broadband between 990 and 1100 cm^−1^ associated with C-O-H bonds is typical of polysaccharides found in seaweed structures [30].

The FT-IR spectra of seaweed film exhibit quite similar results in comparison to filler concentration (1 wt% to 5 wt%), as does the addition of coffee filler into the seaweed matrix. However, certain spectral changes were observed in the wavelength range 1000–1500 cm^−1^. Figure 4 clearly shows the overlap variations in the FT-IR spectra of modified seaweed/coffee film at various filler loadings. This band shifted slightly to a high intensity peak at around 1220 cm^−1^ after the incorporation of coffee with the seaweed, which was attributed to the stretching vibration of the amine group. The presence of an N-H stretching peak indicates that amine functionality was successfully incorporated over the surface of the seaweed film. As the amount of coffee filler in the film increases, so does the intensity of the N-H bond. Overall, the spectra of the seaweed/coffee biopolymer film showed a similar pattern of bands, indicating that coffee and the seaweed polymer matrix are miscible.

**Figure 5:**
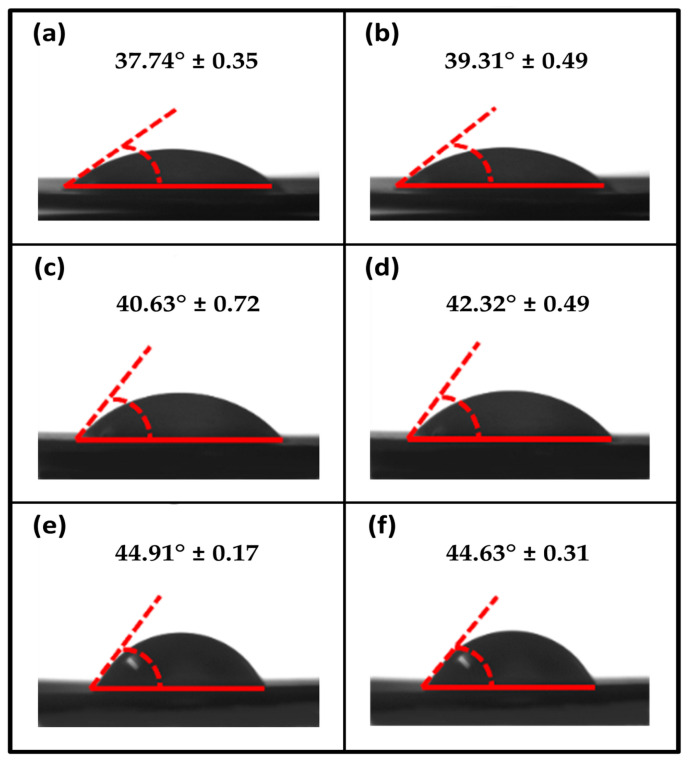
Water contact angle (WCA) of seaweed/coffee biopolymer film at different coffee concentrations: (**a**) control, (**b**) coffee 1 wt%, (**c**) coffee 2 wt%, (**d**) coffee 3 wt%, (**e**) coffee 4 wt% and (**f**) coffee 5 wt%.

The surface wettability of the biopolymer films was determined from the WCA analysis. Figure 5a–f displays a variety of seaweed/coffee biopolymer films at coffee filler concentrations ranging from 0 to 5 wt% by weight. According to Figure 5, seaweed film (control) has the lowest WCA value of all samples tested, 37.74°. This low contact angle value is due to the natural hydrophilic properties of seaweed [29]. However, as the coffee amount increased from 1 to 5 wt%, the WCA of the seaweed film gradually increased from 39.31° to 44.63°. This decrease is most likely due to the hydrophobicity of coffee powder [31]. It is critical for the barrier properties of seaweed/coffee film to have a high WCA value. A high WCA value seaweed film would be more resistant to moisture and water. In packaging applications, this would benefit from a variety of uses while also providing good protection in wet conditions. As previously stated in previous water-related testing, the addition of coffee filler in the polymer matrix improves water barrier properties. When compared to a neat seaweed film, the seaweed film with coffee improves hydrophobicity significantly. In this study, the coffee-modified seaweed had a significantly higher water WCA value than the control film. This demonstrated that the coffee immobilized on the surface of the seaweed film increased the hydrophobicity of the seaweed film.

**Figure 6:**
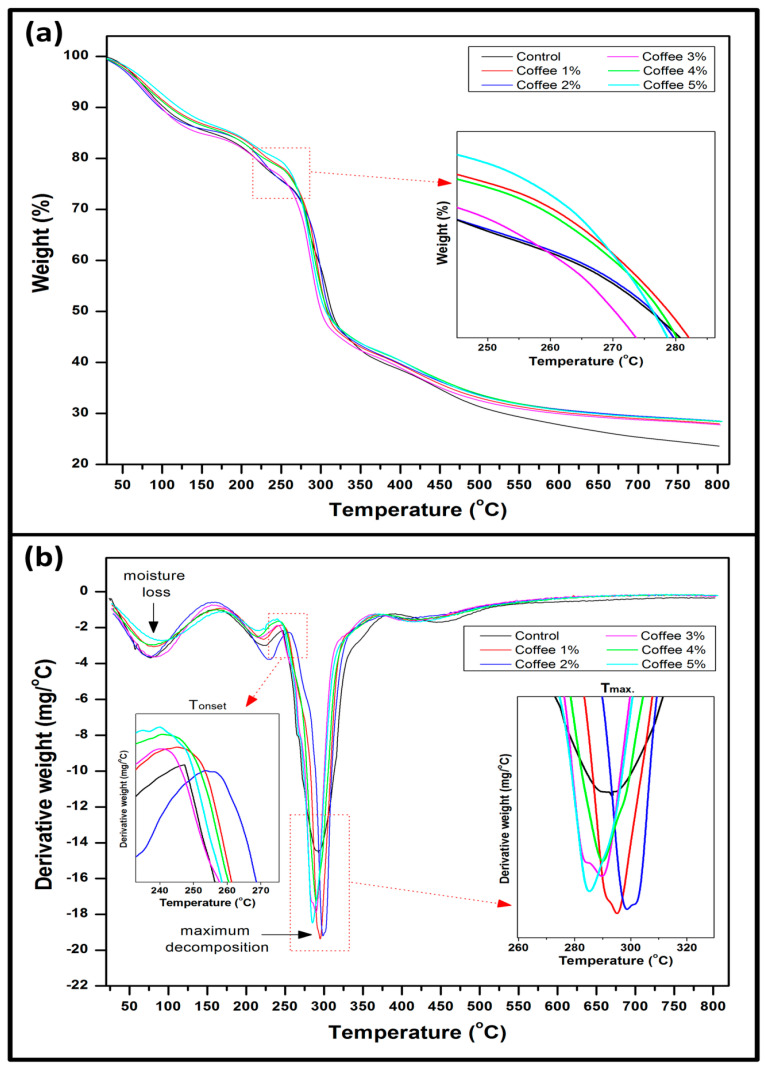
(**a**) Thermogravimetric (TGA) and (**b**) derivative thermogravimetric (DTG) curves of seaweed/coffee biopolymer film at different coffee loadings.

Furthermore, the thermal properties of the films were obtained from the temperature profile of the TGA/DTG curves. The result of the thermogravimetry analysis of the neat seaweed and seaweed/coffee biopolymer is presented in Figure 6. The TGA and DTG curves of seaweed films reinforced with coffee filler are shown in Figure 6a,b, with coffee loadings ranging from 0% to 5% wt. Figure 6a depicts the three thermal degradation phases of all seaweed/coffee films. The initial regions at 50–90 °C (100 °C) were caused by moisture evaporation from the films, which accounted for approximately 10% of the total weight loss of the seaweed film [32]. Seaweed is a naturally hydrophilic biopolymer that absorbs water from its surroundings. The weight loss/thermal decomposition that occurred during this phase was attributed to the evaporation of moisture trapped in the seaweed/coffee biopolymer films. The second step of thermal decomposition of the composite seaweed film begins at around 150 °C and reaches a maximum at around 250 °C, accounting for about 10% of weight loss for all seaweed/coffee composite films. This thermal decomposition could be attributed to the volatilization of glycerol, which was added to the seaweed film as a plasticizer [33]. The major decomposition or third decomposition step for seaweed/coffee films occurred between 212 and 310 °C, which could be attributed to the degradation of the seaweed molecule’s saccharide structure and dehydration of saccharide rings [34]. The decomposition in this region is caused by sulfur dioxide leaving and carbohydrate backbone fragmentation of carrageenan in seaweed, which resulted in film sample decomposition/weight loss [34]. Prior research has shown that the removal of moisture and volatile substances in seaweed biopolymer occurs similarly to the results obtained in this study [32]. These two degradations are not the primary degradation because they were also observed in the control sample, which contained no coffee filler. Previous research has also reported that the degradation of the main seaweed composite films occurring during the third degradation step was in the same range as in this study [10,35]. However, coffee-modified seaweed films have a wider range of thermal degradations as well as a higher onset temperature, indicating greater thermal stability than neat seaweed films (control). The thermal stability of the modified film samples improved after coffee was added to the polymer matrix [36]. The increase in hydrophobicity of the film caused by the coffee filler loading may have an effect on the thermal stability of the seaweed film.

The three stages were confirmed by the derivative thermogravimetric analysis, as shown by the graph, which shows three distinct sets of peaks (Figure 6b). The first shallow peak is due to moisture removal, while the second shallow peak is due to glycerol vaporization. According to Table 1, the lowest onset degradation temperature for control films with 0% coffee filler loading is 257 °C. According to these findings, the addition of coffee reinforcement resulted in a slight increase in degradation temperature. The highest onset degradation temperature for modified films was 286 °C for seaweed film with 5% coffee filler loading. The results show that adding coffee filler to the polymer matrix can lower the weight loss ratio and raise the onset thermal decomposition temperature (T_onset_) of the seaweed films. Strong hydrogen bonding interactions between seaweed and coffee, as well as homogeneous dispersion of the coffee filler in the seaweed matrix, may contribute to the thermal stability of seaweed/coffee composite films in a synergistic manner. Despite this, the increased thermal decomposition temperature in reinforced seaweed composite films was also responsible for the presence of glycerol as seaweed dispersion agents, which promotes better filler and matrix interaction [37]. The addition of filler materials improves the thermal stability of seaweed, which is one of its limitations in packaging applications. The thermal stability of seaweed/coffee composite films improves as 5% coffee filler content was added into the film.

**Figure 7:**
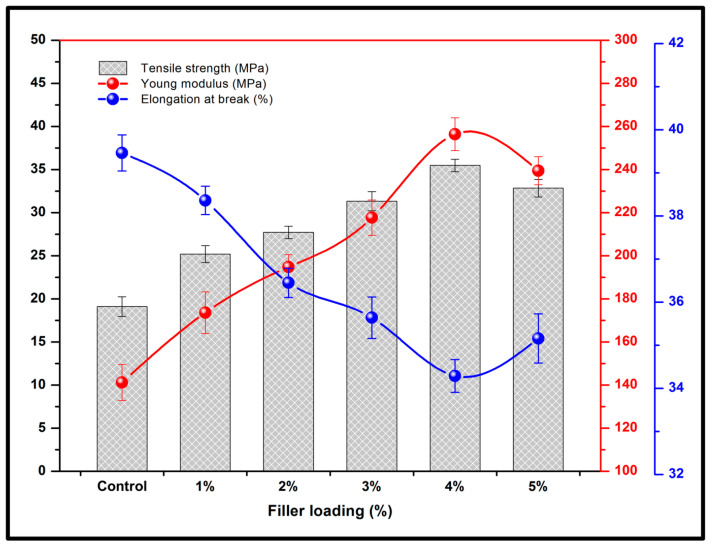
Tensile properties of the fabricated seaweed/coffee biopolymer film.

The tensile test was conducted, and the variation in tensile strength, elongation at break and tensile modulus are illustrated in Figure 7. The biopolymer films indicated increased tensile strength as the incorporated coffee increased. The films exhibited optimum tensile strength (35.47 MPa) as the film was enhanced with the addition of 4 wt% concentration coffee powder. The improved tensile strength was attributed to the good coffee dispersion in the seaweed matrix. The increase in tensile strength indicates that the coffee reinforcement was well dispersed within the polymer matrix, which influenced the adhesive interaction of the coffee reinforcement in the seaweed matrices [38]. The increase in tensile properties was primarily due to coffee’s high surface area, which provided good interfacial bonding between the polymer matrix, resulting in better stress transmission and elastic deformation [39]. Although the tensile strength was reduced after further increases in coffee powder in the matrix network, it was suggested that beyond 3 wt% coffee exhibited decreases in the tensile value (32.84 MPa). This may be attributed to the lack of interfacial adhesion between the coffee filler and the seaweed biopolymer matrix [30]. Interfacial adhesion occurs when two different materials are combined, blended, or mixed. This combination may create a better dispersion of materials into the matrices. Usually, to achieve better interfacial adhesion, the combination of materials must have the same properties, such as hydrophilic fillers and hydrophilic matrices or hydrophobic and hydrophobic materials, which create a strong bond between both materials. As hydrophobic fillers and hydrophobic matrices combine, better dimensional stability can be achieved, but when hydrophilic matrices and hydrophilic fillers or hydrophilic matrices and hydrophobic materials are combined, this may create a problem in the dimensional stability of the composites that are produced [40]. Generally, the wettability increases as the interfacial adhesion increases. However, at the highest 5 wt% coffee, the wettability of the film decreases. As mentioned earlier in the wettability study, the coffee waste material has hydrophobic properties [41]. This might be one of the reasons why the wettability decreased at the highest loading of coffee particles.

Furthermore, in Figure 7, the films exhibited a progressive increased tensile modulus from 141.24 to 256.41 MPa as the composition of coffee powder in the matrix increased from 0 to 4 wt%. Physically, the films demonstrated improved flexibility and good modulus, indicating effective interfacial interactions between the coffee in the seaweed matrix. This suggested that the chemical compounds of the coffee increased the compatibility between the coffee and the seaweed matrix. The enhanced tensile properties of the films with improved tensile strength and tensile modulus could be attributed to the good dispersion of the coffee powder in the seaweed matrix. However, increasing the coffee concentration to 5 wt% resulted in a slight decrease in tensile modulus (239.42 MPa). This leads to poor interfacial bonding between the seaweed matrix and the coffee filler, resulting in reduced mechanical properties. On the contrary to tensile strength and Young’s modulus, the flexibility of the biopolymer films determined by the elongation at break (EAB) decreased linearly with the increase in the coffee content presented in Figure 7. The EAB of the film decreased from 39.46% to 34.29% as the coffee content increased from 0 to 4 wt%. This may be caused by the rigid nature of the filler as previously discussed in the SEM results. The reduction of EAB values with the incorporation of coffee fillers indicates the decreased flexibility of the seaweed films, probably due to strong interactions between the seaweed and coffee fillers and the well dispersion of coffee fillers in the seaweed matrix. Because of the strong interactions between the fillers and the biopolymer matrix, the incorporation of coffee restricts the mobility of the seaweed matrix [41]. However, with increasing coffee filler loadings (5 wt%), the EAB values of seaweed films tend to slightly increased (35.16%), most likely related to the development of agglomeration of the coffee filler in the seaweed matrix [42]. Aggregation formation weakens the interaction between seaweed and the coffee filler, resulting in enhanced flexibility and reduced tensile strength and moduli of the films.

## 4. Conclusions

In this study, seaweed biopolymer films reinforced with varying amounts of coffee filler were successfully fabricated using the solvent casting technique. The incorporation of coffee filler within the polymer matrix improved the physical, chemical, morphological, mechanical, and thermal properties of the seaweed films. The particle size analyzer confirmed that the diameter range of coffee particles was 1106 to 1281 nm. The results show that the particles were well dispersed and distributed, with no aggregation. The negative zeta potential value (−27.0 mV) indicates that the filler has enough mutual repulsion to avoid agglomeration during biopolymer film fabrication. Higher filler dispersion capability is critical for improving the mechanical strength of the film. The rheological properties obtained in this study provide a foundation for correlating the film-forming solution properties to the characteristics of the produced biopolymer film. SEM analysis of the morphological study revealed that coffee fillers were evenly dispersed in seaweed film, which improved the structural properties of the film, most likely due to the excellent compatibility of both materials. The stretching vibration of the O-H, C-H, C=O, S=O, C-N, C-O-C, and C=C groups is indicated by the FT-IR spectra of coffee waste and the seaweed/coffee film. The modification of seaweed films with coffee waste improved their qualities significantly by decreasing the hydrophilicity of the seaweed. Water contact angle (WCA) results show that as the amount of coffee filler increases, so does the hydrophobicity of the seaweed-modified film. Hydrophobic films are required in packaging applications to maintain the original functionality of the film under wet and high humidity conditions. Furthermore, the thermal stability of the biopolymer films was improved after coffee was added to the seaweed polymer matrix. The improvement in mechanical properties of seaweed films was attributed to the strong hydrogen bonding formed between the hydroxyl groups in seaweed and coffee, as determined by FT-IR analysis. The improved mechanical properties of seaweed films are also due to the excellent compatibility of coffee filler with a seaweed matrix and the homogeneous dispersion of coffee fillers in the biopolymer film. In conclusion, the development of a seaweed/coffee biopolymer film is a promising approach for diversifying and adding value to the use of seaweed and coffee waste while also reducing food packaging waste.

## Figures and Tables

**Table 1:** Major thermal decomposition step of seaweed/coffee biopolymer films.

Coffee Filler	Temperature (T_onset_)(ºC)	Char Residue (%)
0%	257	23.59
1%	270	27.98
2%	271	28.52
3%	274	27.76
4%	278	28.41
5%	286	28.46
